# Venereal Disease Problems

**Published:** 1921-02-12

**Authors:** 


### THE PUBLIC WELL-BEING.

### VENEREAL DISEASE PROBLEMS.

### AN EXAMPLE OF FALSE MORALITY.

LAST week we referred to the stubborn and ill-informed attitude of certain religious bodies in regard to the attempts which are now being made by various local authorities to spread abroad a knowledge of the evil consequences of venereal disease, and of the means available for prevention. We instanced in particular the protests raised by certain organisations and individuals in Manchester both against the provision of facilities by the Public Health Committee for early disinfection, and against the wording of certain underground lavatory posters, because they did not lay sufficient stress on the moral side of the question. We are sorry to see that since that article was published the Manchester City Council has agreed to remove the offending clauses in the posters. We are sorry, not so much because of the importance of the posters, but because of the principle at stake. The clause chiefly complained of ran as follows:

> Men who are determined to disregard their moral obligations should read the leaflet "Directions to men," which may be obtained by men on personal application to the Medical Officer of Health, 1 Mount Street, Manchester.

It is not necessary to repeat the reason we have already given against withholding such information. They were well stated at a meeting of the Manchester City Council, at which the subject was discussed at great length. Nobody answered the question put by Councillor Hart: "Is it the business of the Health Committee to prevent contagious disease or to wait until it has developed?" Alderman Jackson, the chairman of the Health Committee, pointed out that their scheme had the full approval of the Ministry of Health, and made the very pertinent observation that if the Council sanctioned the deletion of the clauses complained of in the poster it was tantamount to saying: "We consent to the provision of the centres, but for goodness sake don't let anybody know about it." Miss Margaret Ashton, a social reformer whose knowledge and experience and lofty principles are known throughout the country, urged the vital need of the Manchester treatment centres, the results of which are being watched with the keenest interest by all medical officers in the country, reminded the objectors that the infantile birth-rate is larger because of the spread of venereal disease, and that it is too late to save either mother or child when the disease is contracted, and added these words of common-sense: "We do know that some people are overcome by their passions. When they have fallen are we entitled to give them the chance to save their wives and children? Are we to make it impossible for them to retrace their steps?"

Let us, in addition, recapitulate some figures recently published by the People's League of Health in an attempt to rouse the nation to a sense of its moral responsibilities. The Royal Commission on Venereal Disease estimates that 10 per cent. of all inhabitants of the large towns in England and Wales have been at some time or other infected with syphilis. In the British fighting forces 400,000 casualties from venereal disease would be a moderate computation. All cases of general paralysis of the insane and more than 25 per cent. of the blindness in the country are due to this disease. In face of such terrible figures and the notorious conditions that prevail everywhere, it is permissible to ask whether the fastidious moralists are really on the side of the angels.

